# Crystal structure of (*E*)-9-(4-nitro­benzyl­idene)-8,9-di­hydro­pyrido[2,3-*d*]pyrrolo­[1,2-*a*]pyrimidin-5(7*H*)-one

**DOI:** 10.1107/S2056989016003583

**Published:** 2016-03-04

**Authors:** Khamid U. Khodjaniyazov, Jamshid M. Ashurov

**Affiliations:** a S. Yunusov Institute of the Chemistry of Plant Substances, Academy of Sciences of Uzbekistan, Mirzo Ulugbek Str. 77, Tashkent 100170, Uzbekistan; b A. S. Sadikov Institute of Bioorganic Chemistry, Academy of Sciences of Uzbekistan, Mirzo Ulugbek Str. 83, Tashkent 100125, Uzbekistan

**Keywords:** crystal structure, pyrido­pyrimidine, pyrido­pyrrolo­pyrimidine, 4-nitro­benzaldehyde, yl­idene derivative, hydrogen bonding

## Abstract

The title compound, C_17_H_12_N_4_O_3_, a pyrido­pyrrolo­pyrimidine derivative, is almost planar. The nitro­benzene ring is inclined to the mean plane of the 8,9-di­hydro­pyrido[2,3-*d*]pyrrolo­[1,2-*a*]pyrimidin-5(7*H*)-one moiety (r.m.s. deviation = 0.023 Å) by 6.8 (1)°. In the crystal, mol­ecules are linked *via* C—H⋯O and C—H⋯N hydrogen bonds, forming layers parallel to (101).

## Abstract 2

The title pyrido­pyrrolo­pyrimidine derivative is almost planar, with the benzene ring of the 4-nitro­benzyl­idene substituent inclined to the mean plane of the 8,9-di­hydro­pyrido[2,3-*d*]pyrrolo­[1,2-*a*]pyrimidin-5(7*H*)-one moiety by 6.8 (1)°.

## Chemical context   

Pyrido[2,3-*d*]pyrimidines, and their derivatives, are an important group of heterocyclic compounds that exhibit biological and pharmacological activities. For example, Le Corre *et al.* (2010 ▸) have produced a library of pyrido[2,3-*d*]py­rimi­dines designed as inhibitors of FGFR3 tyrosine kinase. Ramana Reddy *et al.* (2014 ▸) have shown that such compounds are potent inhibitors of cyclin-dependent Kinase 4 (CDK4) and AMPK-related Kinase 5 (ARK5). A series of pyrazolo [*4*,3-*d*]pyrimidin-7-ones were synthesizied to study their pyrido kinases (CDKs) inhibitory activities (Geffken *et al.* 2011 ▸). The anti­tumor activity of some new pyrido[2,3-*d*][1,2,4]triazolo[4,3-*a*]pyrimidin-5-one derivatives have also been studied (El-Nassan, 2011 ▸), and the anti­tumor activity of pyrido[2,3-*d*]pyrimidine and pyrido[2,3-*d*][1,2,4]triazolo[4,3-*a*]pyrimidine derivatives that induce apoptosis through G1 cell-cycle arrest have been reported on by Fares *et al.* (2014 ▸). The above observations prompted us to synthesize the title compound, which contains a pyrido[2,3-*d*]pyrimidin-4-one moiety, and we report herein on its crystal structure.
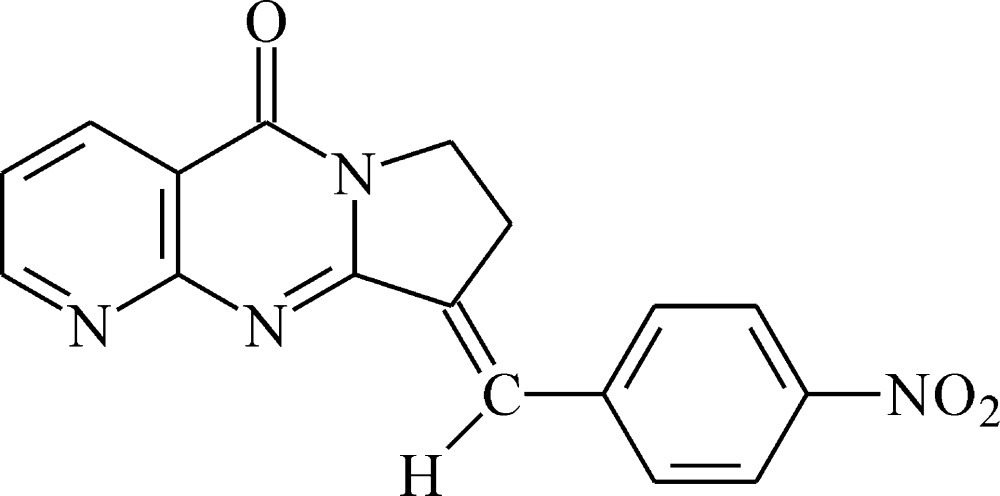


## Structural commentary   

**Figure 1:**
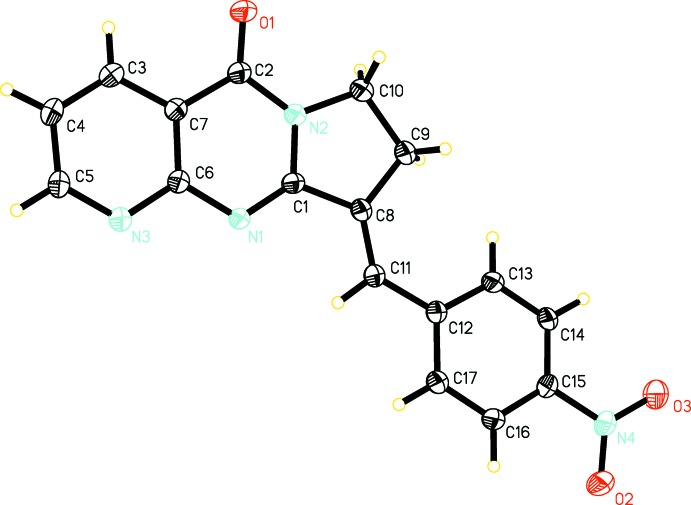
Mol­ecular structure of the title compound, showing the atom labelling. Displacement ellipsoids are drawn at the 50% probability level.

In the mol­ecular structure of the title compound (Fig. 1 ▸), the three fused rings of the 8,9-di­hydro­pyrido[2,3-*d*]pyrrolo[1,2-*a*]pyrimidin-5(7*H*)-one moiety (N1–N3/C1–C10), are essentially planar (r.m.s. deviation = 0.023 Å), with the maximum deviation from the mean plane being 0.036 (2) Å for atom C8. The nitro­benzene ring (C12–C17) is inclined to this mean plane by 6.8 (1)°, while the nitro group (N4/O2/O3) is inclined to the benzene ring by 15.0 (3)°.

## Supra­molecular features   

**Figure 2:**
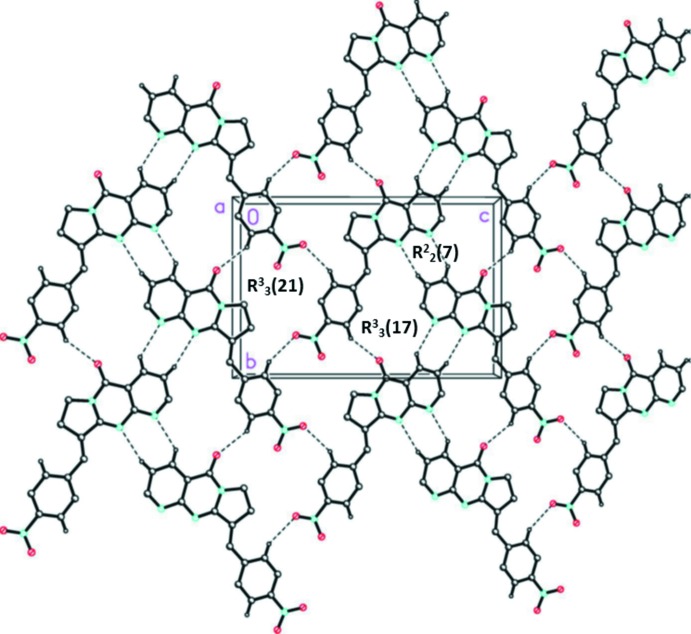
A view along the *a* axis of the crystal packing of the title compound. The hydrogen bonds are shown as dashed lines (see Table 1 ▸). For clarity, H atoms not involved in hydrogen bonding have been omitted.

**Figure 3:**
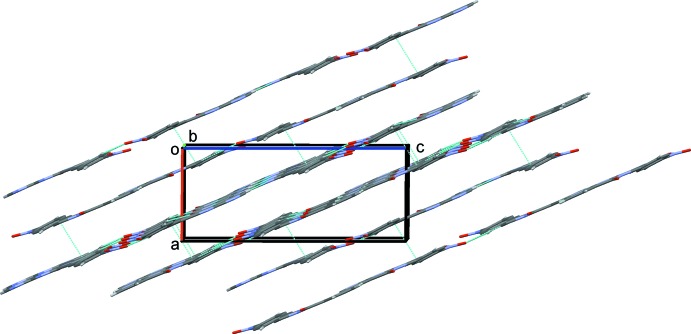
A view along the *b* axis of the crystal packing of the title compound. The hydrogen bonds and inter­planar distances (of *ca* 3.4 Å) are shown as dashed lines (see Table 1 ▸). For clarity, H atoms not involved in hydrogen bonding have been omitted.

In the crystal, mol­ecules are linked *via* C—H⋯O and C—H⋯N hydrogen bonds, forming layers lying parallel to (101); see Fig. 2 ▸ and Table 1 ▸. Within the layers there are 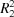
(7), 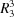
(17), and 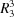
(21) graph-set motifs present (Fig. 2 ▸). The layers are separated by an average inter­planar distance of *ca* 3.4 Å, but there are no significant inter­layer inter­actions present (Fig. 3 ▸).

## Database survey   

**Figure 4:**
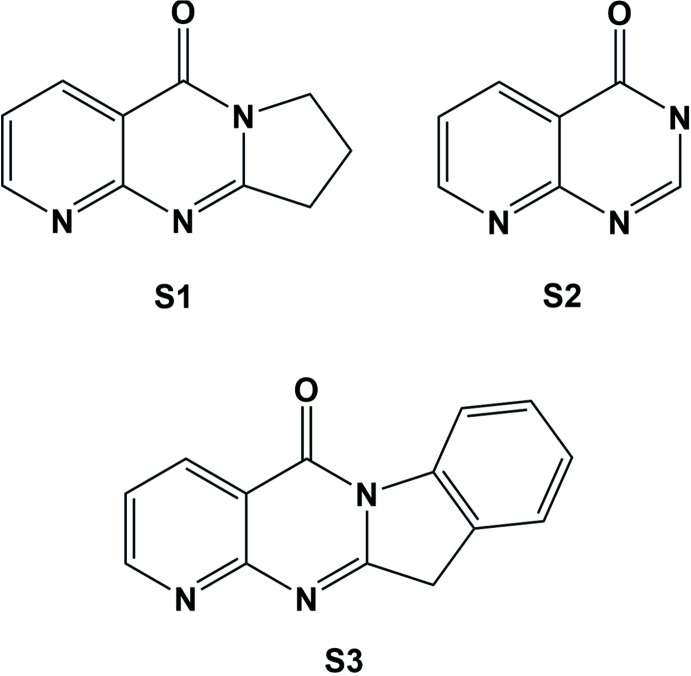
Substructures used for the database survey.

A search of the Cambridge Structural Database (Version 5.37, update November 2015; Groom & Allen, 2014 ▸) was carried out for various substructures (**S1** and **S2**; Fig. 4 ▸) resembling the title compound. For substructure **S1** (8,9-di­hydro­pyrido[2,3-*d*]pyrrolo­[1,2-*a*]pyrimidin-5(7*H*)-one), no hits were obtained. For substructure **S2** (4*H*-3λ2-pyrido[2,3-*d*]pyrimidin-4-one), seven hits were found. Two of these compounds have substructure **S3** (pyrido[2′,3′:4,5]pyrimido[1,2-*a*]indol-5(11*H*)-one), *viz* 9-fluoro­pyrido[2′,3′:4,5]pyrimido[1,2-*a*]indole-5,11-dione (refcode NIJYIP; CCDC 269950; Hicks *et al.*, 2005 ▸), and 9-bromo­pyrido[2′,3′:4,5]pyrimido[1,2-*a*]indole-5,11-dione (refcode NIJYOV; CCDC 218226; DiTusa, 2003 ▸). They are classed as tryptanthrins, which have been shown to have strong anti­bacterial activity, for example, against malaria (Hicks *et al.*, 2005 ▸).

## Synthesis and crystallization   

To a mixture of 2,3-tri­methyl­enepyrido[2,3-*d*]pyrimidin-4-one (0.094 g, 0.5 mmol) and *p*-nitro­benzaldehyde (0.094 g, 0.6 mmol) was added acetic acid (3 ml, 98%). This mixture was refluxed in an oil bath (ca. 423-433 K) for 5 h after which it was left to stand for 24 h. During this time a yellow precipitate formed. It was filtered and washed with distilled water, giving yellow crystals of the title compound (yield: 0.144 g, 0.45 mmol, 90%; m.p. 567–568 K). Yellow block-like crystals suitable for X-ray analysis were grown from a solution of ethanol:water (2:1) by slow evaporation at room temperature. The title product is insoluble in benzene, chloro­form, acetic acid, acetone, DMF, and DMSO, but soluble in tri­fluoro­acetic acid. ^1^H NMR (400MHz, CDCl_3_, δ, p.p.m., *J*/Hz): 3.15 (2H, *td*, *J* = 6.5; 2.9, β-CH_2_), 4.16 (2H, *t*, *J* = 6.5, γ-CH_2_), 7.44 (2H, *d*, *J* = 8.8, H-2′,6′), 7.60 (1H, *dd*, *J* = 7.9; 5.9, H-6), 7.83 (1H, *t*, *J* = 2.9, =CH), 7.98 (2H, *d*, *J* = 8.8, H-3′,5′), 8.63 (1H, *dd*, *J* = 5.9; 1.7, H-5), 9.00 (1H, *dd*, *J* = 7.9; 1.7, H-7). *R*_f_ = 0.47 (chloro­form:methanol, 10:1).

## Refinement   

Crystal data, data collection and structure refinement details are summarized in Table 2 ▸. H atoms were placed in calculated positions and included in the final cycles of refinement using a riding-model approximation: C—H = 0.93–0.97 Å with *U*_iso_(H) = 1.2*U*_eq_(C).

## Supplementary Material

Crystal structure: contains datablock(s) I, global. DOI: 10.1107/S2056989016003583/su5283sup1.cif

Structure factors: contains datablock(s) I. DOI: 10.1107/S2056989016003583/su5283Isup2.hkl

Supporting information file. DOI: 10.1107/S2056989016003583/su5283Isup3.cml

CCDC reference: 1456732

Additional supporting information:  crystallographic information; 3D view; checkCIF report

## Figures and Tables

**Table 1:** Hydrogen-bond geometry (Å, °)

*D*—H⋯*A*	*D*—H	H⋯*A*	*D*⋯*A*	*D*—H⋯*A*
C3—H3⋯N3^i^	0.93	2.58	3.292 (3)	133
C4—H4⋯N1^i^	0.93	2.57	3.480 (3)	166
C13—H13⋯O3^ii^	0.93	2.51	3.363 (3)	153
C16—H16⋯O1^iii^	0.93	2.45	3.259 (3)	145

Symmetry codes: (i) 
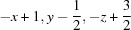
; (ii) 
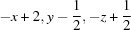
; (iii) 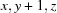.

**Table 2:** Experimental details

Crystal data	
Chemical formula	C_17_H_12_N_4_O_3_
*M*_r_	320.31
Crystal system, space group	Monoclinic, *P*2_1_/*c*
Temperature (K)	293
*a*, *b*, *c* (Å)	7.1755 (3), 11.5855 (3), 17.2515 (5)
β (°)	90.360 (3)
*V* (Å^3^)	1434.12 (8)
*Z*	4
Radiation type	Cu *K*α
μ (mm^−1^)	0.88
Crystal size (mm)	0.20 × 0.18 × 0.15

Data collection	
Diffractometer	Oxford Diffraction Xcalibur Ruby
Absorption correction	Multi-scan (*CrysAlis PRO*; Oxford Diffraction, 2009 ▸)
*T*_min_, *T*_max_	0.928, 1.000
No. of measured, independent and observed [*I* > 2σ(*I*)] reflections	10375, 2965, 2194
*R*_int_	0.045
(sin θ/λ)_max_ (Å^−1^)	0.629

Refinement	
*R*[*F* ^2^ > 2σ(*F* ^2^)], *wR*(*F* ^2^), *S*	0.057, 0.175, 1.08
No. of reflections	2965
No. of parameters	217
H-atom treatment	H-atom parameters constrained
Δρ_max_, Δρ_min_ (e Å^−3^)	0.31, −0.20

Computer programs: *CrysAlis PRO* (Oxford Diffraction, 2009 ▸), *SHELXS97*, *XP* and *SHELXTL* (Sheldrick, 2008 ▸), *SHELXL2014* (Sheldrick, 2015 ▸) and *Mercury* (Macrae *et al.*, 2008 ▸).

## supplementary crystallographic information

### Crystal data

**Table:**

C_17_H_12_N_4_O_3_	*F*(000) = 664
*M**_r_* = 320.31	*D*_x_ = 1.483 Mg m^−^^3^
Monoclinic, *P*2_1_/*c*	Cu *K*α radiation, λ = 1.54184 Å
*a* = 7.1755 (3) Å	Cell parameters from 2842 reflections
*b* = 11.5855 (3) Å	θ = 4.6–75.6°
*c* = 17.2515 (5) Å	µ = 0.88 mm^−^^1^
β = 90.360 (3)°	*T* = 293 K
*V* = 1434.12 (8) Å^3^	Block, yellow
*Z* = 4	0.20 × 0.18 × 0.15 mm

### Data collection

**Table:**

Oxford Diffraction Xcalibur Ruby diffractometer	2194 reflections with *I* > 2σ(*I*)
Detector resolution: 10.2576 pixels mm^-1^	*R*_int_ = 0.045
ω scans	θ_max_ = 76.0°, θ_min_ = 4.6°
Absorption correction: multi-scan (*CrysAlis PRO*; Oxford Diffraction, 2009)	*h* = −8→9
*T*_min_ = 0.928, *T*_max_ = 1.000	*k* = −7→14
10375 measured reflections	*l* = −21→20
2965 independent reflections	

### Refinement

**Table:**

Refinement on *F*^2^	0 restraints
Least-squares matrix: full	Hydrogen site location: inferred from neighbouring sites
*R*[*F*^2^ > 2σ(*F*^2^)] = 0.057	H-atom parameters constrained
*wR*(*F*^2^) = 0.175	*w* = 1/[σ^2^(*F*_o_^2^) + (0.0826*P*)^2^ + 0.3825*P*] where *P* = (*F*_o_^2^ + 2*F*_c_^2^)/3
*S* = 1.08	(Δ/σ)_max_ < 0.001
2965 reflections	Δρ_max_ = 0.31 e Å^−^^3^
217 parameters	Δρ_min_ = −0.20 e Å^−^^3^

### Special details

**Table:**

Geometry. All e.s.d.'s (except the e.s.d. in the dihedral angle between two l.s. planes) are estimated using the full covariance matrix. The cell e.s.d.'s are taken into account individually in the estimation of e.s.d.'s in distances, angles and torsion angles; correlations between e.s.d.'s in cell parameters are only used when they are defined by crystal symmetry. An approximate (isotropic) treatment of cell e.s.d.'s is used for estimating e.s.d.'s involving l.s. planes.

### Fractional atomic coordinates and isotropic or equivalent isotropic displacement parameters (Å^2^)

**Table:**

	*x*	*y*	*z*	*U*_iso_*/*U*_eq_	
O1	0.7005 (3)	−0.09081 (15)	0.54727 (12)	0.0677 (6)	
O2	0.9284 (3)	0.88109 (16)	0.33072 (13)	0.0718 (6)	
O3	0.9755 (5)	0.7632 (2)	0.23673 (14)	0.0913 (9)	
N1	0.6413 (3)	0.24377 (15)	0.62105 (12)	0.0462 (5)	
N2	0.7312 (3)	0.10238 (16)	0.52922 (11)	0.0446 (5)	
N3	0.5181 (4)	0.18637 (18)	0.73866 (13)	0.0584 (6)	
N4	0.9345 (3)	0.78291 (19)	0.30362 (13)	0.0560 (6)	
C1	0.7062 (3)	0.21345 (18)	0.55449 (13)	0.0428 (5)	
C2	0.6831 (4)	0.00730 (19)	0.57260 (15)	0.0471 (5)	
C3	0.5521 (4)	−0.0476 (2)	0.70117 (16)	0.0527 (6)	
H3	0.5655	−0.1253	0.6891	0.063*	
C4	0.4772 (4)	−0.0150 (2)	0.76967 (16)	0.0572 (7)	
H4	0.4362	−0.0698	0.8051	0.069*	
C5	0.4631 (5)	0.1021 (2)	0.78581 (16)	0.0611 (7)	
H5	0.4114	0.1232	0.8330	0.073*	
C6	0.5898 (3)	0.15420 (19)	0.66978 (14)	0.0454 (5)	
C7	0.6088 (3)	0.03714 (19)	0.64897 (13)	0.0441 (5)	
C8	0.7641 (3)	0.29244 (19)	0.49255 (13)	0.0428 (5)	
C9	0.8364 (4)	0.2207 (2)	0.42615 (14)	0.0506 (6)	
H9A	0.9678	0.2356	0.4178	0.061*	
H9B	0.7685	0.2379	0.3787	0.061*	
C10	0.8050 (4)	0.0949 (2)	0.45079 (15)	0.0526 (6)	
H10A	0.7167	0.0572	0.4164	0.063*	
H10B	0.9213	0.0522	0.4503	0.063*	
C11	0.7470 (3)	0.4070 (2)	0.50057 (14)	0.0452 (5)	
H11	0.6939	0.4308	0.5469	0.054*	
C12	0.7990 (3)	0.50060 (19)	0.44770 (13)	0.0422 (5)	
C13	0.8910 (4)	0.4833 (2)	0.37725 (15)	0.0502 (6)	
H13	0.9231	0.4089	0.3621	0.060*	
C14	0.9345 (4)	0.5759 (2)	0.33012 (14)	0.0495 (6)	
H14	0.9938	0.5643	0.2830	0.059*	
C15	0.8886 (3)	0.6858 (2)	0.35404 (13)	0.0459 (5)	
C16	0.8010 (4)	0.7066 (2)	0.42325 (14)	0.0474 (5)	
H16	0.7732	0.7816	0.4385	0.057*	
C17	0.7552 (3)	0.6140 (2)	0.46970 (14)	0.0465 (5)	
H17	0.6944	0.6270	0.5163	0.056*	

### Atomic displacement parameters (Å^2^)

**Table:**

	*U*^11^	*U*^22^	*U*^33^	*U*^12^	*U*^13^	*U*^23^
O1	0.1034 (16)	0.0362 (9)	0.0640 (12)	−0.0012 (9)	0.0249 (11)	−0.0063 (8)
O2	0.1033 (17)	0.0415 (10)	0.0708 (13)	−0.0056 (10)	0.0169 (12)	0.0069 (9)
O3	0.151 (2)	0.0669 (14)	0.0568 (13)	0.0001 (14)	0.0446 (15)	0.0151 (10)
N1	0.0638 (12)	0.0332 (9)	0.0419 (10)	−0.0014 (8)	0.0163 (9)	−0.0002 (7)
N2	0.0558 (11)	0.0365 (9)	0.0416 (10)	0.0003 (8)	0.0106 (8)	−0.0027 (7)
N3	0.0854 (16)	0.0414 (11)	0.0485 (12)	0.0009 (10)	0.0222 (11)	0.0014 (9)
N4	0.0722 (14)	0.0476 (11)	0.0485 (12)	−0.0036 (10)	0.0120 (10)	0.0120 (9)
C1	0.0499 (12)	0.0336 (10)	0.0448 (12)	0.0002 (8)	0.0075 (9)	−0.0014 (8)
C2	0.0582 (13)	0.0356 (11)	0.0476 (13)	−0.0007 (9)	0.0096 (10)	−0.0010 (9)
C3	0.0683 (15)	0.0383 (11)	0.0516 (14)	−0.0024 (11)	0.0052 (12)	0.0048 (10)
C4	0.0755 (17)	0.0467 (13)	0.0496 (14)	−0.0040 (12)	0.0113 (12)	0.0123 (11)
C5	0.087 (2)	0.0504 (14)	0.0460 (14)	0.0001 (13)	0.0224 (13)	0.0072 (11)
C6	0.0562 (13)	0.0387 (11)	0.0414 (11)	0.0005 (9)	0.0095 (10)	0.0026 (9)
C7	0.0517 (12)	0.0374 (11)	0.0433 (12)	−0.0003 (9)	0.0073 (10)	0.0014 (9)
C8	0.0496 (12)	0.0422 (12)	0.0368 (11)	−0.0013 (9)	0.0102 (9)	−0.0022 (9)
C9	0.0649 (14)	0.0481 (12)	0.0389 (12)	−0.0027 (11)	0.0120 (10)	−0.0025 (10)
C10	0.0688 (16)	0.0437 (12)	0.0455 (13)	−0.0001 (11)	0.0116 (12)	−0.0053 (10)
C11	0.0559 (13)	0.0409 (11)	0.0390 (11)	0.0001 (9)	0.0124 (9)	0.0010 (9)
C12	0.0498 (12)	0.0401 (11)	0.0369 (11)	−0.0017 (9)	0.0083 (9)	0.0012 (8)
C13	0.0695 (15)	0.0378 (11)	0.0435 (13)	0.0005 (10)	0.0154 (11)	−0.0026 (9)
C14	0.0687 (15)	0.0463 (12)	0.0335 (11)	−0.0020 (11)	0.0165 (10)	−0.0013 (9)
C15	0.0558 (13)	0.0405 (11)	0.0415 (12)	−0.0039 (9)	0.0040 (10)	0.0074 (9)
C16	0.0604 (14)	0.0377 (11)	0.0440 (12)	0.0016 (9)	0.0095 (10)	−0.0019 (9)
C17	0.0552 (13)	0.0438 (12)	0.0406 (12)	0.0019 (10)	0.0115 (10)	−0.0006 (9)

### Geometric parameters (Å, º)

**Table:**

O1—C2	1.225 (3)	C8—C11	1.340 (3)
O2—N4	1.231 (3)	C8—C9	1.510 (3)
O3—N4	1.214 (3)	C9—C10	1.535 (3)
N1—C1	1.291 (3)	C9—H9A	0.9700
N1—C6	1.387 (3)	C9—H9B	0.9700
N2—C1	1.371 (3)	C10—H10A	0.9700
N2—C2	1.377 (3)	C10—H10B	0.9700
N2—C10	1.459 (3)	C11—C12	1.467 (3)
N3—C5	1.332 (3)	C11—H11	0.9300
N3—C6	1.350 (3)	C12—C13	1.401 (3)
N4—C15	1.461 (3)	C12—C17	1.404 (3)
C1—C8	1.469 (3)	C13—C14	1.383 (3)
C2—C7	1.466 (3)	C13—H13	0.9300
C3—C4	1.355 (4)	C14—C15	1.379 (3)
C3—C7	1.394 (3)	C14—H14	0.9300
C3—H3	0.9300	C15—C16	1.374 (3)
C4—C5	1.389 (4)	C16—C17	1.380 (3)
C4—H4	0.9300	C16—H16	0.9300
C5—H5	0.9300	C17—H17	0.9300
C6—C7	1.410 (3)		
			
C1—N1—C6	115.76 (19)	C8—C9—H9A	110.7
C1—N2—C2	123.0 (2)	C10—C9—H9A	110.7
C1—N2—C10	113.55 (19)	C8—C9—H9B	110.7
C2—N2—C10	123.40 (19)	C10—C9—H9B	110.7
C5—N3—C6	116.8 (2)	H9A—C9—H9B	108.8
O3—N4—O2	123.0 (2)	N2—C10—C9	104.79 (18)
O3—N4—C15	118.5 (2)	N2—C10—H10A	110.8
O2—N4—C15	118.5 (2)	C9—C10—H10A	110.8
N1—C1—N2	125.9 (2)	N2—C10—H10B	110.8
N1—C1—C8	125.7 (2)	C9—C10—H10B	110.8
N2—C1—C8	108.39 (19)	H10A—C10—H10B	108.9
O1—C2—N2	121.5 (2)	C8—C11—C12	130.1 (2)
O1—C2—C7	125.3 (2)	C8—C11—H11	114.9
N2—C2—C7	113.18 (19)	C12—C11—H11	114.9
C4—C3—C7	119.1 (2)	C13—C12—C17	118.4 (2)
C4—C3—H3	120.4	C13—C12—C11	123.8 (2)
C7—C3—H3	120.4	C17—C12—C11	117.8 (2)
C3—C4—C5	118.5 (2)	C14—C13—C12	120.5 (2)
C3—C4—H4	120.8	C14—C13—H13	119.7
C5—C4—H4	120.8	C12—C13—H13	119.7
N3—C5—C4	124.8 (3)	C15—C14—C13	119.0 (2)
N3—C5—H5	117.6	C15—C14—H14	120.5
C4—C5—H5	117.6	C13—C14—H14	120.5
N3—C6—N1	115.5 (2)	C16—C15—C14	122.3 (2)
N3—C6—C7	121.8 (2)	C16—C15—N4	119.2 (2)
N1—C6—C7	122.6 (2)	C14—C15—N4	118.5 (2)
C3—C7—C6	118.9 (2)	C15—C16—C17	118.7 (2)
C3—C7—C2	121.6 (2)	C15—C16—H16	120.7
C6—C7—C2	119.5 (2)	C17—C16—H16	120.7
C11—C8—C1	121.0 (2)	C16—C17—C12	121.1 (2)
C11—C8—C9	131.0 (2)	C16—C17—H17	119.5
C1—C8—C9	108.02 (19)	C12—C17—H17	119.5
C8—C9—C10	105.11 (19)		
			
C6—N1—C1—N2	−1.3 (4)	N1—C1—C8—C11	−2.2 (4)
C6—N1—C1—C8	178.0 (2)	N2—C1—C8—C11	177.2 (2)
C2—N2—C1—N1	2.0 (4)	N1—C1—C8—C9	178.3 (2)
C10—N2—C1—N1	179.3 (2)	N2—C1—C8—C9	−2.4 (3)
C2—N2—C1—C8	−177.4 (2)	C11—C8—C9—C10	−175.8 (3)
C10—N2—C1—C8	−0.1 (3)	C1—C8—C9—C10	3.7 (3)
C1—N2—C2—O1	177.2 (3)	C1—N2—C10—C9	2.5 (3)
C10—N2—C2—O1	0.2 (4)	C2—N2—C10—C9	179.7 (2)
C1—N2—C2—C7	−1.9 (4)	C8—C9—C10—N2	−3.7 (3)
C10—N2—C2—C7	−178.9 (2)	C1—C8—C11—C12	178.5 (2)
C7—C3—C4—C5	−1.2 (5)	C9—C8—C11—C12	−2.1 (5)
C6—N3—C5—C4	1.1 (5)	C8—C11—C12—C13	−4.0 (4)
C3—C4—C5—N3	0.0 (5)	C8—C11—C12—C17	176.6 (3)
C5—N3—C6—N1	178.4 (3)	C17—C12—C13—C14	−1.1 (4)
C5—N3—C6—C7	−1.0 (4)	C11—C12—C13—C14	179.6 (3)
C1—N1—C6—N3	−178.6 (2)	C12—C13—C14—C15	1.0 (4)
C1—N1—C6—C7	0.8 (4)	C13—C14—C15—C16	0.0 (4)
C4—C3—C7—C6	1.2 (4)	C13—C14—C15—N4	−179.7 (2)
C4—C3—C7—C2	−177.2 (3)	O3—N4—C15—C16	−165.0 (3)
N3—C6—C7—C3	−0.1 (4)	O2—N4—C15—C16	14.5 (4)
N1—C6—C7—C3	−179.4 (2)	O3—N4—C15—C14	14.8 (4)
N3—C6—C7—C2	178.4 (2)	O2—N4—C15—C14	−165.7 (3)
N1—C6—C7—C2	−1.0 (4)	C14—C15—C16—C17	−1.0 (4)
O1—C2—C7—C3	0.8 (4)	N4—C15—C16—C17	178.8 (2)
N2—C2—C7—C3	179.9 (2)	C15—C16—C17—C12	0.9 (4)
O1—C2—C7—C6	−177.7 (3)	C13—C12—C17—C16	0.1 (4)
N2—C2—C7—C6	1.4 (3)	C11—C12—C17—C16	179.5 (2)

### Hydrogen-bond geometry (Å, º)

**Table:**

*D*—H···*A*	*D*—H	H···*A*	*D*···*A*	*D*—H···*A*
C3—H3···N3^i^	0.93	2.58	3.292 (3)	133
C4—H4···N1^i^	0.93	2.57	3.480 (3)	166
C13—H13···O3^ii^	0.93	2.51	3.363 (3)	153
C16—H16···O1^iii^	0.93	2.45	3.259 (3)	145

Symmetry codes: (i) −*x*+1, *y*−1/2, −*z*+3/2; (ii) −*x*+2, *y*−1/2, −*z*+1/2; (iii) *x*, *y*+1, *z*.
